# Autologous bone fragments for skull reconstruction after microvascular decompression

**DOI:** 10.1186/s12893-022-01820-8

**Published:** 2022-11-18

**Authors:** Yuankun Cai, Xiuling Zhang, Xiaobin Chen, Xuan Dai, Songshan Chai, Guo Li, Zhimin Mei, Joshua Ho, Jincao Chen, Luoqing Li, Nanxiang Xiong

**Affiliations:** 1 Department of Neurosurgery, Zhongnan Hospital of Wuhan University, Wuhan, Hubei China; 2 Department of Neurology, Xiaogan Hospital Affiliated to Wuhan University of Science and Technology, Xiaogan, Hubei China; 3 Department of Neurosurgery, Wuhan NO. 1 Hospital, Wuhan, Hubei China; 4 School of Biomedical Sciences, LKS Faculty of Medicine, Hongkong University, Hongkong, China; 5 Department of Neurology, Yueyang Central Hospital, Yueyang, Hunan China

**Keywords:** Microvascular decompression, MVD, Skull Reconstruction, Autologous bone fragments, Long-term outcome

## Abstract

**Background:**

Various methods are used to reconstruct the skull after microvascular decompression, giving their own advantages and disadvantages. The objective of this study was to evaluate the efficacy of using autologous bone fragments for skull reconstruction after microvascular decompression.

**Methods:**

The clinical and follow-up data of 145 patients who underwent microvascular decompression and skull reconstruction using autologous bone fragments in our hospital from September 2020 to September 2021 were retrospectively analyzed.

**Results:**

Three patients (2.06%) had delayed wound healing after surgery and were discharged after wound cleaning. No patient developed postoperative cerebrospinal fluid leakage, incisional dehiscence, or intracranial infection. Eighty-five (58.62%) patients underwent follow-up cranial computed tomography at 1 year postoperatively, showed excellent skull reconstruction. And, the longer the follow-up period, the more satisfactory the cranial repair. Two patients underwent re-operation for recurrence of hemifacial spasm, and intraoperative observation revealed that the initial skull defect was filled with new skull bone.

**Conclusion:**

The use of autologous bone fragments for skull reconstruction after microvascular decompression is safe and feasible, with few postoperative wound complications and excellent long-term repair results.

## Background

Microvascular decompression (MVD) was first proposed by Jannetta [1] and has become the most common surgical procedure for various cranial neurovascular compression syndromes [2, 3]. The surgery is usually done using a suboccipital retrosigmoid approach, with a small bone flap craniotomy performed in most cases [4–6]. Because an incomplete skull is associated with postoperative complications such as cerebrospinal fluid leakage, postoperative skull reconstruction is required even for small bone window craniotomy [7]. Materials used to repair cranial defects after MVD usually include autologous bone flaps and artificial biomaterials [8–10]. Surgeons favor autologous bone flaps because of their excellent histocompatibility [11, 12]. However, fixation of autogenous bone flaps still requires a metal coupling piece and often results in bone resorption, especially in younger patients [13, 14].

Three artificial biomaterials are available for skull reconstruction: cement pastes, osteoactive biomaterials, and prefabricated polymers [15]. Each has advantages and disadvantages: cement pastes don’t induce the formation of new bone, osteoactive biomaterials allow for the induction of bone formation, while polymers allow for vascular and bone growth without resorption [7]. Although introducing various new biomaterials has brought more options for cranial repair, it almost always increases the cost of care [16].

To our knowledge, there has been no report of cranial repair after MVD using autologous bone fragments, except for our team [17]. The Centre has consistently used the autogenous bone to repair post-MVD bone window defects and has performed long-term follow-up. Therefore, the present study retrospectively analyzed the data of patients who underwent MVD and subsequent skull reconstruction with autologous bone fragments at our center and reported on their postoperative wound complications and long-term outcomes of skull reconstruction.

## Methods

Institutional Review Board/Ethics Committee approval was not required for this retrospective analysis of de-identified medicare data. Likewise, patient consent was not applicable to our study. The basic characteristics, imaging data, intraoperative findings, and postoperative management of the included patients were reviewed. A total of 145 patients who underwent MVD and skull reconstruction using autologous bone fragments were included in the study. The basic characteristics of the patients and diseases are summarized in Table 1.

**Table 1:** Patient basic characteristics and postoperative complications

Characteristics	Value (n = 145)	Ratio (%)
Sex		
Male	49	33.79
Female	96	66.21
Age (y/o)	52.99 ± 9.18	–
Major diagnosis		
HFS	90	62.07
TN	46	31.72
GN	6	4.14
Intractable Tinnitus	2	1.38
INN	1	0.69
Complications		
Delayed wound healing	3	2.07
Incisional dehiscence	0	–
Cerebrospinal fluid leakage	0	–
Intracranial infection	0	–
Length of postoperative stay	6.01 ± 0.83	–

*HFS* hemifacial spasm, *TN* trigeminal neuralgia, *GN* glossopharyngeal neuralgia, *INN* intermediate nerve neuralgia

### Operative technique

Under general anesthesia, the patient was placed in the lateral position, with the head facing toward the opposite side. A vertical scalp incision of approximately 5 cm through the “star point” was created, and the muscles and connective tissue were separated to reveal the skull surface. After drilling a bur hole with a 9-mm electric drill (Hitachi, Tokyo, Japan), craniectomy was performed using rongeurs to enlarge the diameter of the bur hole to 2 cm. The resultant skull fragments were collected and preserved in a special container.

**Fig. 1:**
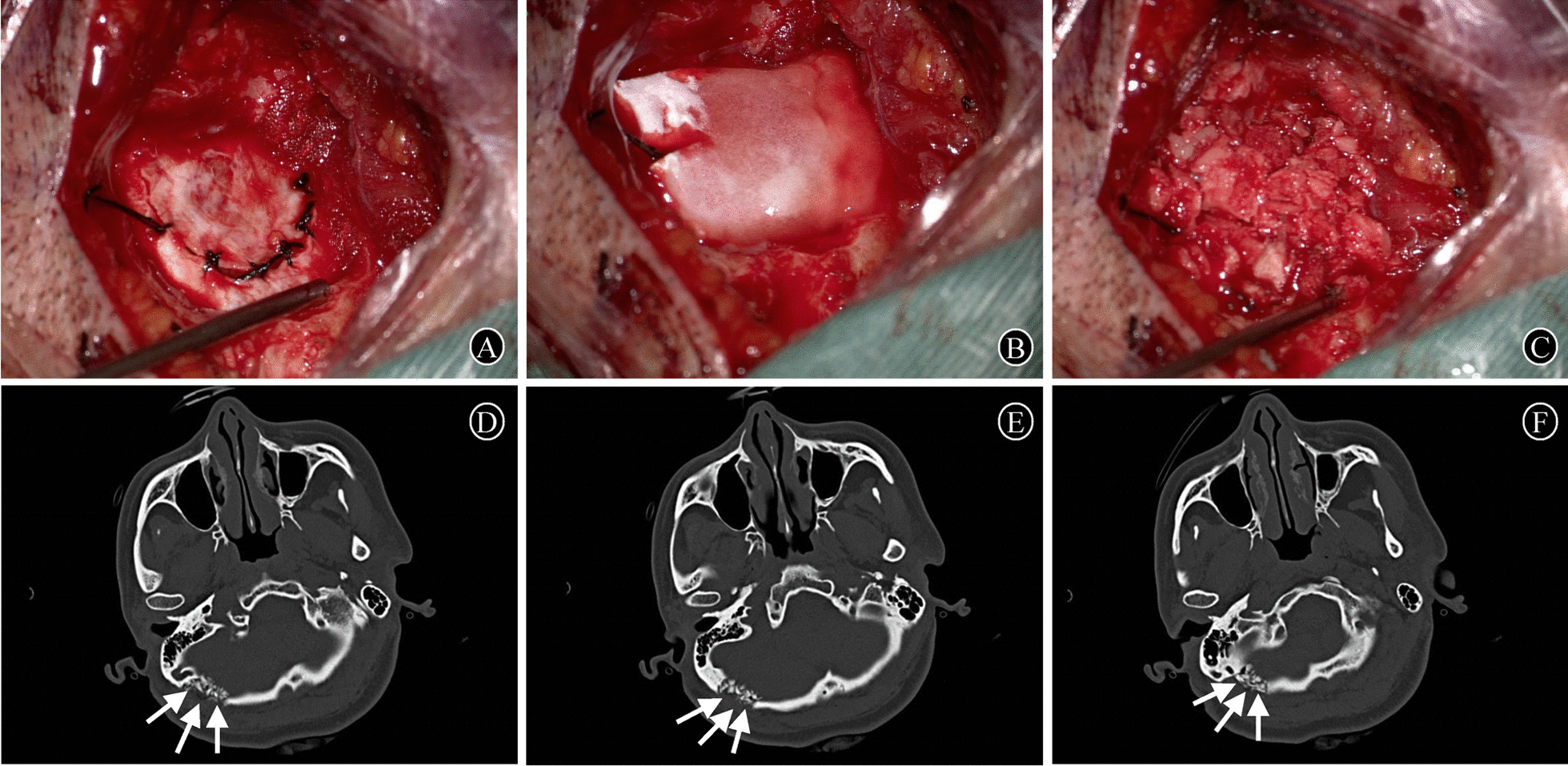
Intraoperative procedure of skull reconstruction and cranial CT results on the first postoperative day. **A** The dura was closed with watertight sutures after completion of the intracranial microvascular decompression procedure. **B** A layer of gelatin sponge was placed over the sutured dura. **C** The autologous skull fragments were placed so that they evenly covered the gelatin sponge. **D**, **E**, **F** The patient’s cranial CT on the first postoperative day showed that the autologous bone fragments filled in the skull defect evenly (white arrow)

The dura was closed with watertight sutures after the neurovascular decompression operation was completed and it was confirmed that there was no significant intracranial bleeding (Fig. 1A). Next, a layer of gelatin sponge matching the size of the skull defect was placed over the sutured dura (Fig. 1B). The autologous skull fragments collected during craniotomy were placed so that they evenly covered the gelatin sponge (Fig. 1 C). Finally, the muscle, subcutaneous tissue, and scalp were tightly closed in a layer-by-layer manner.

### Postoperative management and follow-up

The patients underwent a cranial computed tomography (CT) examination on the first postoperative day to exclude postoperative intracranial hemorrhage and check for bone fragment filling (Fig. 1D–F). The wound was checked by a dedicated person from the first postoperative day until discharge, focusing on the presence of wound redness, swelling, and dehiscence.

All patients included in the study underwent two telephone follow-up, 1 month after surgery and at the beginning of this study. Patients were advised to undergo clinical visits, examinations, and cranial CT in the outpatient clinic one year after discharge to check the skull reconstruction. Follow-up focus includes not only focusing on wound healing and cranial repair but also the clinical effects of MVD surgery.

## Results

The procedure was completed successfully and safely in all patients, with a mean operative time of 2.14 ± 0.32 h. No mortality or other severe intra- and postoperative complications were observed. Three patients (2.06%) had delayed wound healing after surgery and were discharged after wound cleaning. No patient developed postoperative cerebrospinal fluid leakage, incisional dehiscence, or intracranial infection. Telephone follow-up data obtained at 1 month after discharge showed that no patient had significant wound complications, including the three patients with delayed wound healing during hospitalization.

**Fig. 2:**
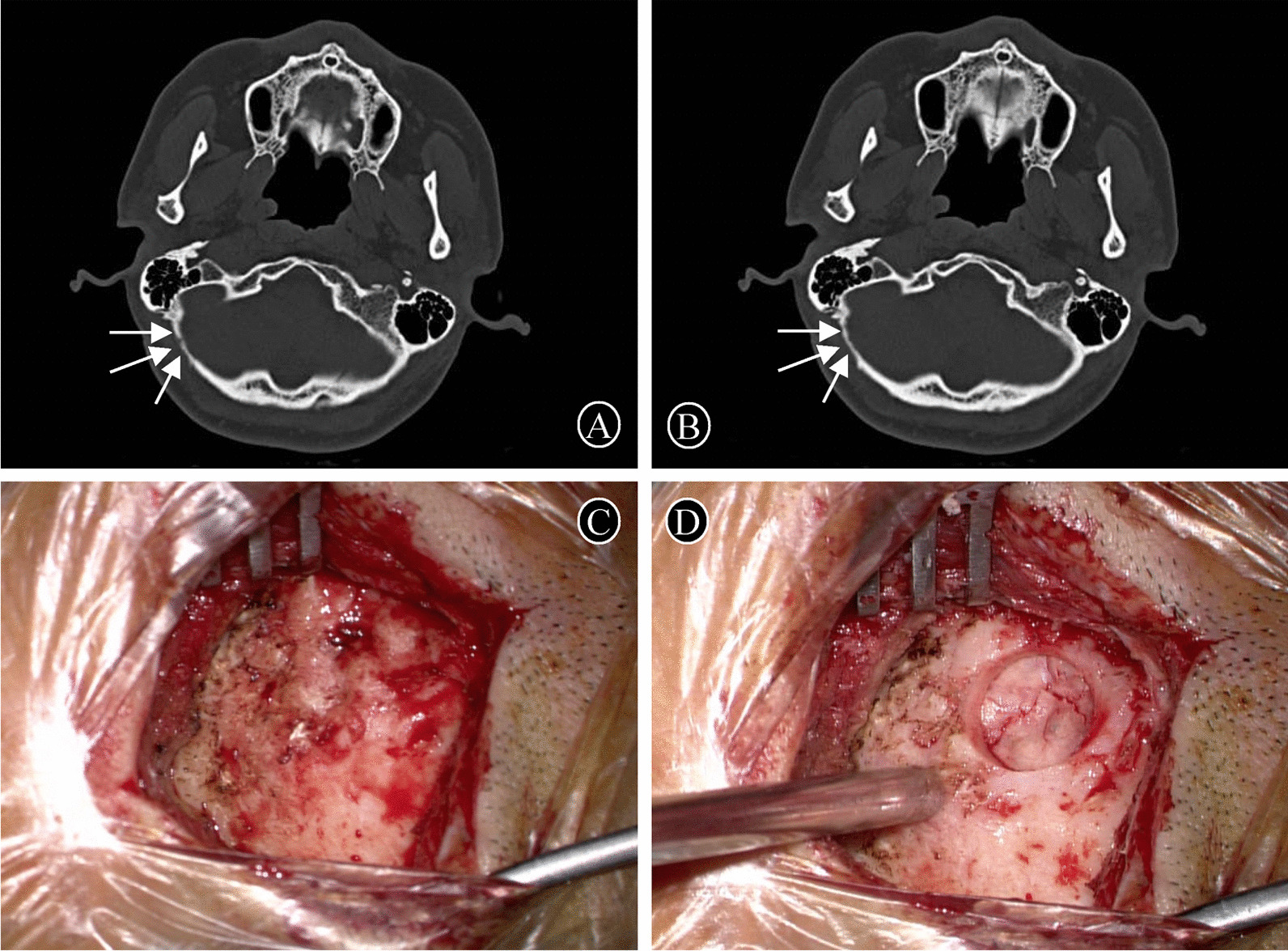
Cranial CT and intraoperative findings at one year after skull reconstruction. **A**, **B** The skull defect was completely reconstructed on cranial CT one year after the skull reconstruction (white arrow). **C**, **D** A second MVD one year later showed that the new skull bone filled the skull defect caused by the previous operation

At the beginning of this study, we followed up all patients again by telephone. The mean last follow-up time after the telephone interview was 11.3 ± 3.19 months (Table 1). The surgical effectiveness of MVD reached 87.59% (n = 127), and no patient developed wound-related complications during follow-up. Two patients were recommended to be readmitted for MVD surgery after 1 and 1.5 years postoperatively because of recurrence of facial spasm, respectively. The second craniotomy showed that the new skull bone had replaced the autologous bone fragments placed during the first surgery (Fig. 2C, D).

A total of 85 patients returned to our hospital approximately 1 year after surgery for clinical visits, examinations and cranial CT. Clinical visits and examinations revealed that all patients had healed incisions, no significant pain on compression, and palpable skull formation. Skull CT results of different patients showed that all autologous bone fragments had been wholly resorbed. Instead, new bone grows in varying degrees of concentration from around the skull defect until it fills it (Fig. 2 A, B). The remaining patients reported no discomfort at the surgical incision site, which may be why they did not return to the hospital for a repeat cranial CT.

## Discussion

With the advancement of microneurosurgical techniques, the size of the bone window required for MVD surgery is gradually decreasing. Studies have reported that a small bone window of about 2 cm is adequate for MVD surgery and obtains the same surgical outcomes as a large bone window [18, 19]. In some institutions, a small bone flap is milled out with a milling tool and fixed with a connecting piece; in others, the bone window is enlarged with a rongeurs and repaired postoperatively with titanium mesh or other artificial bone repair material. However, all of the above methods involve the implantation of a foreign body in the skull and some additional medical costs. Our team used rongeurs to enlarge the bone window during MVD craniotomy and used the collected bone fragments for cranial reconstruction after MVD [17]. It not only avoids the need for foreign body implantation, but also reduces medical costs.

The present study retrospectively analyzed the wound healing after MVD and skull reconstruction using autologous bone fragments in our center. The results showed that only 2.07% of patients had delayed healing, which is lower than the complication rate of 3.7–14.2% reported in other studies [20, 21]. In our opinion, this low complication rate is primarily attributable to the excellent histocompatibility characteristics of autologous bone fragments, which are unmatched by any other allogeneic material [11, 12, 16]. Although an increasing number of allogeneic materials with minimal resistance are being developed and used for skull reconstruction, the occurrence of immune resistance and infection after implantation, which necessitates secondary surgery, is still unavoidable [22, 23].

Secondly, the good results achieved in the present study are also associated with the manipulation in our skull reconstruction. The autologous bone fragments were evenly placed on the epidural gelatin sponge, which not only avoided the occurrence of cerebrospinal fluid leakage, but also effectively reduced the residual wound cavity between the bone window and the dura. This wound cavity is unfavorable for hemodynamic reconstruction at the site of the cranial defect and tends to form aseptic inflammation, increasing the risk of wound complications. Therefore, the reduction of the wound cavity by filling with autologous bone fragments may be another important reason for the lower wound complications in the present study compared with other studies.

In the long-term follow-up of the present study, cranial CT showed that new skull bone had replaced the autologous bone fragments, with complete skull reconstruction at the site of the skull defect. Although the exact mechanism of skull reconstruction by autologous bone fragments is not clear, we believe that it may be associated with functions of bone grafts: osteoconduction, osteoinduction, and osteogenesis [24, 25]. The autologous bone fragments are implanted in close contact with the bone window edge, stimulating the formation of new vessels towards the skull defect site. The relatively loose bone fragments increase the surface area and favor the hemodynamic reconstruction of the skull defect site, providing a favorable environment for skull reconstruction. In addition, stimulation of the autologous bone fragments causes periosteal osteogenesis at the edge of the bone window and extends towards the defect site, which is known as osteoconduction.

Osteoinduction is the process by which mesenchymal stem cells at and around the host site are recruited to differentiate into chondroblasts and osteoblasts. Various inducible proteins and growth factors are recruited through reconstructed hemodynamics to promote bone fragment demineralization, and the demineralized cartilage is then subjected to induced osteogenesis. This dynamic process of bone fragment resorption and remodeling is known as the “crawling replacement" [26–28].

Osteogenesis describes the process in which cells from bone fragments survive the transfer to the defect site and form new bone, which is critical in the initial phase of bone repair [29, 30]. Studies have shown that autologous bone fragments contain many active osteoblasts that could be used for skull reconstruction through osteogenesis [31]. In addition, it has also been shown that progenitor cells brought about by hematopoietic reconstruction at the site of the skull defect could also differentiate into osteoblasts for direct osteogenesis [32]. In conclusion, although the mechanism of skull reconstruction by autologous bone fragments remains uncertain, the excellent skull reconstruction outcome deserves clinical promotion.

Combined with the results of this study, we suggest that autologous bone fragments have the following three advantages for repairing cranial defects after MVD. First, the use of a rongeur rather than a milling tool during craniotomy effectively reduces the damage to the dura and sigmoid sinus during the procedure. Secondly, autologous bone fragments fill the overall cranial defect, avoiding the use of other biomaterials including metal connecting pieces, which not only effectively reduces postoperative incision-related complications, but also reduces patient medical costs. Finally, distant cranial CT images and intraoperative findings suggest that the autologous fragmented bone can be resorbed to form new cranial bone for effective cranial repair.

## Conclusion

The use of autologous bone fragments for skull reconstruction after MVD results in few wound complications and achieves excellent long-term cranial repair results. Furthermore, the method is convenient and does not increase medical costs.

## Data Availability

The data that support the findings of this study are available on request from the corresponding author (NX.X).

## Abbreviations

CT: Computed tomography
GN: Glossopharyngeal neuralgia
HFS: Hemifacial spasm
INN: Intermediate nerve neuralgia
MVD: Microvascular decompression
TN: Trigeminal neuralgia
